# Lines to a Delinquent Debtor

**Published:** 1912-02

**Authors:** Ben King


### Lines to a Delinquent Debtor.

If I should die tonight,
And you should come to my cold corpse and say,
Weeping and heart sick, o'er my lifeless clay—
If I should die tonight,
And you should come in deepest grief and woe—
And say, "Here's that ten dollars that I owe,"
I might arise in my large white cravat
And say, "What's that?"

If I should die tonight,
And you should come to my cold corpse and kneel,
Clasping my bier, to show the grief you feel—
I say, if I should die tonight,
And you should come to me, and there and then,
Just even hint 'bout paying me that ten—
I might arise the while,
But I'd drop dead again.

—*Ben King.*

(Respectfully dedicated to some of my delinquent subscribers. Please remit.)

---

SUN YAT SEN, President of the Republic of China, is a doctor. graduate of an English medical college at Hong Kong.

---

DEATH OF AN OLD TEXAS PHYSICIAN.—Dr. Q. C. Smith, for thirty years a practicing physician in Austin, Texas, died at his home in San Diego, Cal., October 28, 1911, of paralysis of the bowels. Dr. Smith removed to San Diego some few years ago. He was a disciple of Bolling and a graduate of the Medical College of Nashville. He was a Tennesseean by birth.

